# The Role of Intersectionality and Context in Measuring Gender-Based Violence in Universities and Research-Performing Organizations in Europe for the Development of Inclusive Structural Interventions

**DOI:** 10.1177/10778012241231773

**Published:** 2024-02-19

**Authors:** Anne Laure Humbert, Sofia Strid, Jagriti Tanwar, Anke Lipinsky, Claudia Schredl

**Affiliations:** 1 Centre for Diversity Policy Research and Practice, 6395 Oxford Brookes University, Oxford, UK; 2 Department of Sociology and Work Science, 3570 University of Gothenburg, Gothenburg, Sweden; 3 Department of OB&HRM, Nottingham University Business School, University of Nottingham, Nottingham, UK; 4 G 28363 ESIS Leibniz Institute for the Social Sciences, Cologne, Germany; 5 28363 GESIS Leibniz Institute for the Social Sciences, Cologne, Germany

**Keywords:** gender-based violence, intersectionality, surveys, research-performing organizations, theorizing quantitative measurement

## Abstract

The aim of the article is to discuss how thinking about gender-based violence intersectionally and in context can not only enrich our understanding but also lead to transformative change in organizations. The article argues that to better understand gender-based violence in universities and research institutions, analyses need to be intersectional and contextual. Such approaches go beyond binary understandings of gender and narrow legalistic definitions of gender-based violence. The article reflects on how to operationalize this to derive starting points for intersectional categories to consider and contextual factors to measure at micro-, meso-, and macro-levels. It concludes that a multilevel intersectional analysis leads to more nuanced knowledge on experiences of gender-based violence and is, therefore, better equipped to inform the development of measures to eradicate the problem in an inclusive way.

## Introduction

To combat and attempt to eradicate gender-based violence, a necessary step is to understand the true scale of the problem, who might be more at risk than others, and the consequences that gender-based violence can have. Creating an empirical measurement instrument to produce evidence on gender-based violence is therefore crucial. Global and European surveys of gender-based violence have been increasingly conducted over the course of the past three decades (Merry, 2016), such as the EU-wide survey on violence against women (European Union Agency for Fundamental Rights, 2014) or Eurobarometer 85.3 on gender-based violence (European Commission, 2018), drawing on and complementing national-level surveys. These usually focus on violence against women and provide valuable comparative data across countries and individual situations. However, comparatively, less is known about gender-based violence in the context of universities and other research organizations even though the issue has been ongoing, but overlooked, or worse as Sharoni and Klocke (2019) argue, covered up.

The topic of gender-based violence has recently attracted greater attention from policymakers, as seen in recent policy development at the EU level. The policy priorities of the European Research Area (ERA) unveiled in 2012 did not originally consider gender-based violence, and instead focused on removing barriers to gender equality in the career progression of researchers; gender balance in decision-making positions; and the inclusion of gender in research content (European Commission, 2012). By 2021, this had changed, and as part of the effort toward “deepening the ERA” (Council of the European Union, 2021b), the topic of gender-based violence was included as one of the priorities under ERA Action 5 seeking to “*Promote gender equality and foster inclusiveness, taking note of the Ljubljana Declaration”* (Council of the European Union, 2021a). In parallel, the announcement by the European Commission that gender equality plans (GEPs) were to become mandatory for universities and other research-performing organizations to remain eligible for EU research funding provided further focus on the issue of gender-based violence. According to the guidelines of the European Commission, gender-based violence is one of the five recommended priority areas which GEPs should address (European Commission, 2021).

The European project “Gender-based violence and institutional responses: Building a knowledge base and operational tools to make universities and research organizations safe” (UniSAFE) responds to the need to obtain evidence (both quantitative and qualitative) and analyze the data in context (organizational and national) to provide better understandings, insights, tools, and methods to combat gender-based violence in research organizations (Strid et al., 2021). The project has implemented a cross-cultural survey of 46 institutions across 15 countries carried out between January 2022 and May 2022 (Lipinsky et al., 2022). However, it is not just data on gender-based violence in universities and other research-performing organizations that are needed, but also better analyses. This cannot be achieved without measuring and analyzing gender-based violence both intersectionally (that is the ways in which multiple inequalities, including gender inequalities, intersect) and in context—and ultimately reveal the structural and institutional dimensions of gender-based violence that may create specific vulnerabilities in research and higher education environments.

This article addresses the conceptual and theoretical approaches that are needed for further improving analyses of gender-based violence, using the context of universities and research organizations, but with relevance to gender-based violence in other organizational contexts. The article starts by defining gender-based violence, the different forms it can take, the lack of clarity as to what it encompasses, and extending its understanding beyond an individual problem and instead one related to a social structure of dominance, with violence itself regarded as an inequality in its own right (Hearn et al., 2022). It then engages with the importance of intersectionality for analyzing gender-based violence, including whether and how to stabilize analytical measurement categories, what sets of social relations to prioritize, and which to consider empirically (Walby et al., 2012). Next, the article introduces the ecological framework approach as a way to expand intersectional analysis by providing depth through context-focused categories, both at meso- and macrolevel (Heise, 1998). It concludes with a discussion section that aims at showing how combining intersectionality and contextual analyses can inform our understanding of potential vulnerabilities to gender-based violence as well as contribute to the development of inclusive structural interventions to eradicate gender-based violence in universities and other types of research organizations.

## The Importance of Defining Gender-Based Violence

### Definitions of Different Forms of Gender-Based Violence Used in Research

According to the European Commission (2022), gender-based violence is defined as: “Violence directed against a person because of that person's gender or violence that affects persons of a particular gender disproportionately.” It is not limited to violence against women, and it may affect all people, though women and minoritized groups are disproportionately affected. Gender-based violence has a polymorphous structure and regroups many forms, which are more or less easy to capture quantitatively (Musso et al., 2020). These different forms include physical, sexual, psychological, and economic violence (Council of Europe, 2011). Physical violence is an easily identifiable form, with incidents such as hitting, slapping, or punching (Heise, 1998; Hester et al., 1996). Sexual violence can relate to physical assault with rape being one of the most serious types of incidents, but also includes incidents that are constitutive of sexual harassment, such as inappropriate jokes or sexual advances (Kelly, 1988; Phipps, 2018). Psychological violence involves bullying, exclusion, incivility, or social/professional undermining (Council of Europe, 2011; Vveinhardt, 2019). Economic violence relates to the unfair withholding of resources in a way that is detrimental to, for example, a career or work (Krigel & Benjamin, 2021; Postmus et al., 2020).

It is also important to consider how gender-based violence is evolving in the context of the digitalization of our world, and the relation between the online and offline forms of violence (Dunn, 2020). The development of the internet and associated information and communication technologies, over the course of a couple of decades, have been nothing short of a socioeconomic revolution. This revolution is marked by a generational effect, with so-called digital natives whose socialization has been shaped by these tools and for whom online spaces are as normal as offline spaces. Given that most of today's university students belong to this cohort, it is essential to consider the effects of new modalities, if not new forms, of gender-based violence. Thus far, few studies have examined this aspect (for an exception, see Faucher et al., 2014), despite the fact that in the digital world, gender-based violence finds “*a new technical capacity to hurt*” (Musso et al., 2020, p. 261).

The focus of the majority of studies on gender-based violence is typically on either intimate-partner violence (a type of gender-based violence defined by the relationship to the perpetrator) or sexual violence (a type of gender-based violence defined by the nature of the incident; Bondestam & Lundqvist, 2020; Bradbury-Jones et al., 2019). However, gender-based violence is not limited to intimate-partner violence, rather it is a much wider and more complex phenomenon that permeates not only the home but also the place of study or work. One of the defining features of intimate-partner violence is that victims and perpetrators “*share a world”* (Musso et al., 2020, p. 265), and gender-based violence that happens in the work or study place can also be understood as reflecting this idea of a shared world or context.

The forms of violence captured in existing surveys matter beyond data collection. Analyses may provide prevalence rates for each form of violence separately, or aggregate them (often subject to checks of the reliability of the scales, e.g., Cronbach’s alpha or confirmatory factor analysis). However, such aggregation needs to be informed by theoretical considerations about what forms to include. For example, the Council of Europe's (2011) four forms of gender-based violence (physical, psychological, sexual, and economic) are neither widely understood nor transposed in research designs by many. In particular, there is low awareness of the concept of economic violence (van Gelder et al., 2021), and lower applicability to a context outside of intimate-partner violence (Postmus et al., 2020). Similarly, another example would be considerations about the extent to which psychological violence is gender-based, and where the boundaries between “universal” and gender-based harassment lie, if such a distinction is possible. This calls for the creation of a strong typology of the forms of gender-based violence, underpinned by a conceptual framework informed by theoretical insights, and validated empirically through statistical methods and data.

### The Need for Greater Conceptual Clarity

Conceptual clarity—and associated terminology—matters particularly in light of the widespread conflation of the terms “gender-based violence” and “violence against women”. Though most incidents of gender-based violence are perpetrated by men against women, and though both the severity and consequences of the violence experienced by women at the hands of men are much greater, not all gender-based violence is perpetrated by men and against women. Further, while measuring gender-based violence against women is a starting point, there is great value in measuring gender-based violence that affects other gender groups than women. This is particularly important as exposure to gender-based violence against nonbinary and trans persons may be comparatively higher (Davidson, 2016; European Union Agency for Fundamental Rights, 2020; Jordan et al., 2020; Voth Schrag, 2017), but also because gender-based violence against men does take place but tends to remain unspoken of and highly stigmatized (Thobejane et al., 2018).

The conflation between “gender-based violence” and “violence against women” is somewhat related to another widespread conflation, between “sex” and “gender.” Sex and gender both operate on a spectrum (Guenther et al., 2018, p. 266). The majority of people are assigned a clear sex (usually at birth), a marker of their biological anatomy and characteristics, as either female or male. A small fraction, however, will fall into the category of intersex, for example, if they present with ambiguous genitalia or are diagnosed with a sex chromosome so-called “anomaly.” Gender is socially constructed on the basis of this initial sex binary categorization; babies are socialized as girls and boys (see Butler, 1993 for a discussion of the “girling the girl” process), and are later expected to conform to the expectations, performance, and practices assigned to the binary categories of women and men. While for most people there is an alignment between sex and gender, there is still a nonnegligible number of people that are somewhere in the middle between these two ends of the artificial gender spectrum. Being nonbinary, being queer, being trans—among many other possible gender identities—is important to recognize precisely because it might be associated with a higher risk of gender-based violence. The expectation that sex and gender expectations correspond to each other extends to sexual orientation, with society structured by the principle of heteronormativity (Butler, 1990). Deviating from this, that is being gay, lesbian, or bisexual—also among many other possible sexual orientations—can represent a significant additional at-risk-factor of experiencing gender-based violence.

## Gender-Based Violence as Part of a Wider System of Dominance and Power

### Inequalities

Gender-based violence can be regarded as a subset of violence (Hearn et al., 2022). It regroups those incidents that disproportionately affect people because of their sex, gender, or sexual orientation (including being female, being a woman, not conforming to hegemonic masculinity ideals, being nonbinary, not being heterosexual, being trans, being queer, etc.) based on exploiting and reifying gender power relations; and feeds from prejudiced and/or discriminatory norms, attitudes and stereotypes in the wider environment (e.g., an organization, a community, or a country). Violence as a whole, unlike gender-based violence, predominantly affects men, and it too tends to respond to gendered patterns, that is, violence as an expression of certain types of masculinities. As such, violence in this more general sense, is most often perpetrated by men over other men. Measuring broader violence also matters, as it allows us to better contextualize and contrast forms of violence that are gender-based, though it must be kept analytically separate (Strid et al., 2021). The distinction between violence and gender-based violence may be blurry. In practical terms, how can we distinguish between different forms, and determine which are “gendered” or “gender-based”? In fact, it is possible that most respondents of online surveys on the topic of gender-based violence are less likely to make this distinction when reflecting on and disclosing their own past experiences.

Gender-based violence is both a cause and a consequence of wider gender inequalities, as well as an inequality in its own right (Hearn et al., 2022). There cannot be gender equality in society without combating and eradicating gender-based violence, and yet, tackling the problem is largely absent hitherto when it comes to making organizations, and more specifically, universities and other research organizations more gender equal. Gender-based violence is endemic, persistent, and adaptive: it exists in a variety of contexts and geographies that reflect the universality of masculine dominance (Musso et al., 2020). Wemrell et al. (2019), drawing on MacKinnon (1979), remind us that gender-based violence can be understood as the product of “layers of dominance,” which responds to expectations of masculinity; the exercising of control over the other; and with the central aim to maintain a social hierarchical order. Dominance differs from power, in that it is a “naturalized” form of power, one that is largely imperceptible and unrecognized even to the dominated groups/individuals; and which is exerted on the basis of an implicit consensus and assumed societal order (Musso et al., 2020).

### Gender-Based Violence in (Research-Performing) Organizations

Gender-based violence is the production and reproduction of a structural gender system that embodies dominance within the structure of organizations. In this system, women and men are defined as social categories, with respective performative expectations of femininities and masculinities. Acts of violence, notably gender-based violence, serve to maintain dominance over women as a group (and other gender groups). This can be understood through the lens of the “gendered organization”: Acker (1990) developed a systematic theory of gender and organization that addressed the need to understand how gender inequalities were in part created through organizational processes and practices; and how organizations produced and reproduced gendered practices. Gender-based violence, as a manifestation of gender inequalities, is thus embedded in this gendered organizational context and serves to maintain power inequalities between groups including those delineated along the axes of sex, gender identity, and/or sexual orientation.

Ignoring the organizational context, or assuming that it is gender-neutral therefore ignores how gender-based violence—notably sexual harassment—is both a cause and consequence of gender inequalities in organizations (MacKinnon, 1979; O’Connor et al., 2021), and not only in society. Further, regarding organizations as gender neutral can also serve to regard incidents of gender-based violence such as sexual harassment as “*deviations of gendered actors [rather than] components of organizational structure*” (Acker, 1990, p. 142 referring to MacKinnon's work, 1979). Organizational contexts differ, including in how they are gendered, in their institutional structures or staff/student composition which is bound to influence the prevalence of gender-based violence and the effectiveness of any measures put in place to combat it.

As Acker (1990) has noted, organizational processes and practices shape gender identities, particularly so masculinity (used as singular in the original text, and therefore attributable to a certain form of masculinity as hegemonic). Gender-based violence is reified by “hegemonic masculinity,” understood as a version of masculinity, a cultural ideal, an aspiration, and as such limited to a small number of men, and which is material in the sense that it is embodied, performative, and realized (Hearn, 2012). As Hearn points out, hegemonic masculinity is performative: it combines ideology and materiality. It is a nebulous concept:

Does it refer to cultural aspirations, representations, everyday practices, or institutional structures? How do various dominant ways of men – respectable (corporate/though/aggressive/violence); controlling resources – interconnect with each other? (Hearn, 2012, p. 590)

The materiality of control, violence, or aggression by men can be located at the individual level, though gender-based violence needs to be understood as a wider ideological system of dominance by men over women (and other gender groups) at the meso- and macrolevel.

The research that has been conducted on gender-based violence in universities and other research organizations tends to depart from the topics in the more general literature on gender-based violence by looking at gender-based violence perpetrated by both intimate partners and nonintimate partners and the issue of sexual harassment and violence (Bondestam & Lundqvist, 2020; Cantor et al., 2020; Coker et al., 2015; Linder & Myers, 2018; Sharoni & Klocke, 2019; Sutherland et al., 2016). Gender-based violence in the context of universities and other research organizations operates partially under its own logic, and just like intimate-partner violence, it is necessary to understand the relational nature between incidents and the context-based/located perpetrators, as well as how incidents relate to the exercising of power relations from some over others, with the aim to produce and reproduce a system of dominance (O’Connor et al., 2021). In the context of universities and other research organizations, status hierarchies, age, and gender hierarchies are intertwined in an environment that serves learning and knowledge production (Naezer et al., 2019).

In this section, we have set out to define our terminology and concept, with the aim to provide a firm foundation for a better analysis of gender-based violence in the context of universities and other research organizations. We have discussed the boundaries of gender-based violence within the more general landscape of violence, to understand how it relates to gendered structural factors located at the micro-, meso-, and macrolevels. We are mindful that the ultimate aim of analyzing gender-based violence is to eradicate it, and that to do so means to understand who is more at risk and what factors might increase those risks. We therefore conceptualize context variables of research institutions as an additional layer of preexisting intersecting inequalities. Both can create situations of vulnerability in which some people are at a higher risk of experiencing gender-based violence, or are at risk of not benefiting from protective measures in place compared with other people in the same environment. We look back at Van Dijk and Steinmetz's (1980) risk model (cited in Van Dijk, 2016), which identifies three key factors: economic/psychological vulnerabilities of the target; proximity/colocation with the perpetrator, for example, a specific location, here that of universities and research organizations; and low social/practical protection against violence, that is for us the context of the gendered organization. Adapting this risk-model to gender-based violence shows the importance of combining both an intersectional lens (addressing potential vulnerabilities) and a contextual lens (understanding how the organizational and national contexts shape experiences of violence). We look at both aspects in turn in the following sections.

## The Importance of Intersectionality in Measuring and Analyzing Gender-Based Violence

### Considerations in Taking an Intersectional Approach to Analyzing Gender-Based Violence

It is recognized that gender-based violence is shaped by structural inequalities besides gender, which create multiple and intersectional forms of discrimination and disadvantage (UN Women, 2011) and call for considering the particular circumstances that can make some people more vulnerable (Council of Europe, 2011). Intersectionality as a term originates from the work of Crenshaw (1989, 1991), even if the concept denoted by the term is much older (Hearn et al., 2016) and can be understood as multiple inequalities shaped by different axes of power among different sets of social relations (Walby et al., 2012). Despite growing recognition of the pertinence of intersectionality in research as well as at the policy level, there is limited research on gender-based violence that addresses intersectionality (Colpitts, 2022; Musso et al., 2020), and this is despite the fact that Crenshaw's original work on intersectionality (Crenshaw, 1991) did focus on violence against women.

Taking an intersectional analytical approach speaks to two aspects. First, it allows to break down experiences of gender-based violence by different groups, according to factors that might create disadvantages and/or vulnerabilities, and without losing sight of the “actions of the powerful” (Walby et al., 2012, p. 228); and second, it also allows us to consider the experiences of gender-based violence beyond those of women alone, and instead extend the analysis to experiences by men and nonbinary people in relation to the prisms of trans status and gender identity. Other grounds of inequalities create positionalities where intersections can aggravate the consequences of gender-based violence, sometimes referred to as the “minority stress” effect in the literature (Nybergh et al., 2016). Minority stress has been described as “*stress resulting from experienced and internalized homophobia”* (Messinger, 2011, p. 2229) in the context of sexual minorities, though this is generalizable to other minoritized groups. For example, being from a minoritized group in relation to sexual orientation or gender identity might mean that experiences of gender-based violence are not recognized or are trivialized by others, and the individuals themselves are not supported or worse, ridiculed. Yet, gender-based violence against some minoritized groups has not often been measured empirically in surveys owing to a lack of operationalization and small sample sizes (Magliozzi et al., 2016). Considering these other groups matters because victims might feel invisible and not worthy of help, with no dedicated support provision in place (e.g., counseling or financial assistance). The first step for more visibility is to be counted, that is obtaining visibility through data. This is only a first step, and care needs to be exercised to ensure that data are available also for those that have the most power, to avoid data on minoritized groups being used to marginalize/essentialize their experiences further, for example, gender-based violence as a “minority issue” (Walby et al., 2012).

Paying attention to the interaction of sex and/or gender with other grounds of diversity means considering questions such as whether sex and/or gender moderate experiences of violence for different groups and what we can learn from the interactions of sex and/or gender with other grounds of diversity among different groups. The existing literature has noted that certain sociodemographic factors can heighten the risk of exposure to various forms of gender-based violence (Voth Schrag, 2017), because of how they are positioned within power relations in their organizations as “others” (O’Connor et al., 2021). Membership in certain minoritized groups is associated with higher exposure to gender-based violence, including in the context of universities and other research organizations. These include gender identity; sexual minorities (Messinger, 2011); ethnic, racial, or cultural minorities (Roudsari et al., 2009; Wemrell et al., 2019); migrants (Gonçalves & Matos, 2020; Keygnaert & Guieu, 2015; Voolma, 2018); younger people (Voth Schrag, 2017), international students (Forbes-Mewett & McCulloch, 2016), precarious contracts (Bondestam & Lundqvist, 2020), among others.

### Challenges in Combining an Intersectional Approach With a Categorical Approach

Reconciling the needs for an intersectional approach with that of the requirements of a quantitative approach is not without challenges. Here McCall's (2005) perspective is useful in that she sees categorical approaches as a way to look at issues among specific groups, particularly “vulnerable” ones (as opposed to anticategorical approaches that allow for an analysis of the salience of group identities in the first place; their boundaries, if any; and criteria for membership of these groups). Addressing inequalities therefore relies on the ability to document and analyze the problem of gender-based violence, both within and between different groups. However, the creation of these discrete “groups” or “categorizations” are in themselves problematic in that there is a risk that they become over-stabilized, and thus unhelpful if this ends up essentializing and reifying differences and social relations (Crenshaw, 1991; McCall, 2005; Walby et al., 2012), and conflate “unity” with group “uniformity” (Hancock, 2007). The question then becomes “*how to balance the stability and fluidity of inequalities so they are sufficiently stable as to be available for empirical analysis, while recognizing that they change”* (Walby et al., 2012, p. 228).

Moving past the tension of whether to regard categories as fluid or stable is nonetheless possible, by considering how categories can be regarded as “heuristic devices” (Cho et al., 2013, p. 786) that can help in analyzing intersecting inequalities, and considering that:

intersectionality is best framed as an analytical sensibility. If intersectionality is an analytic disposition, a way of thinking about and conducting analysis, then what makes an analysis intersectional is not its use of the term ‘intersectionality’ […] Rather, what make an analysis intersectional – whatever term it deploys, whatever its iteration, whatever its field or discipline – is its adoption of an intersectional way of thinking about the problem of sameness and difference and its relation to power. This framing – conceiving of categories not as distinct but as always permeated by other categories, fluid and changing, always in the process of creating and being created by dynamics of power – emphasizes what intersectionality does rather than what intersectionality is. (Cho et al., 2013, p. 795)

A way forward is to follow the advice of Walby et al. (2012, p. 231), who drawing on the work of Ferree (2009) and Choo and Ferree (2010), argue for the need to:

recognize the historically constructed nature of social inequalities and their sedimentations in social institutions. […] At any one moment in time, these relations of inequality have some stability as a consequence of their institutionalization, but over a period of time they do change. The institutionalization of social relations often provides a degree of relative stability to the experience of social inequality.

This pragmatic approach to categorization can enable the shift of emphasis from individuals and their category membership(s), toward an analysis of the social dynamics and relations between individuals, that is from “categories of identity” toward “structure of inequalities” (Cho et al., 2013, p. 797).

A body of literature is increasingly discussing how to transpose the intersectional framework approach into a more advanced quantitative methodology (Bauer et al., 2021; Evans et al., 2018; Merlo, 2018). These works also recognize that social relations are created to correspond, more or less well, to individual identities (including their intersections), and that sets of social relations are shaped by complex systems, imbued by power relations that reflect wider structural inequalities (Hancock, 2007). They seek to find quantitative measures that will provide answers to the following questions (Bauer et al., 2021): How are sets of social relations constituted, and along what axes? How do these sets of social relations reflect interpersonal, often historical, mechanisms of oppression, marginalization, and/or minoritization? How are these sets of social relations shaped by a wider complex system of structural inequalities? Is intersectional disadvantage “additive” or “multiplicative”? Within a quantitative approach, intersectionality therefore ought to be regarded as:

an analytical sensibility, […] a theoretical framework that requires quantitative researchers to avoid assuming heterogeneity across intersections both in outcomes and processes and to structure their research and its interpretation around social power. (Bauer et al., 2021, p. 2)

The operationalization of intersectionality into quantitative methods, to find answers to these questions has tended to involve the use of cross-tabulations, analysis of differences between means or the use of regression models. However, the use of these methods is not without criticisms and calls to improve the quantitative approaches used to answer these questions (Saperstein & Westbrook, 2021). Cross-tabulations, for example, are limited in that they can be disclosive of any intersectional categories with too few individuals, but also in that they fail to account for other relevant variables (Spierings, 2012). Focusing on means is seen as acting to the detriment of showing the heterogeneity within sets of social relations, in what Merlo (2018), for example, has called the “tyranny of averages.” How regression models are specified, that is the variables capturing intersecting inequalities, needs a subtle approach that balances the statistical principle of parsimony with calls to fit more comprehensive models informed by a political specification (Hancock, 2007). Regression models are also seen as problematic if they only incorporate main effects, as this is seen as largely representing an additive approach that is at odds with the theoretical approach of intersectionality (Bauer et al., 2021; Hancock, 2007). Adding fixed effects can reify minoritized positions, by leaving privilege invisible (since typically, for example, it is the categories of “woman” and “Black” that are included rather than “man” or “White”), but also because it assumes that more identities are associated with more harm, even though some identities might work in opposite directions (e.g., to continue with our example, “White woman” or “Black man”; Evans et al., 2018). Of course, it is possible to add interaction terms (Bauer et al., 2021; Hancock, 2007; Spierings, 2012; Weldon, 2006), though traditional modeling is limited in the number of interactions that it can consider.

### Multilevel Approaches to Conducting Intersectional Analyses of Gender-Based Violence

Recent methodological work has shown how multilevel models could be used to take intersectionality into account, commonly referred to as the multilevel analysis of individual heterogeneity and discriminatory accuracy (MAIHDA) in the literature (Evans et al., 2018; Merlo, 2018). This approach has two useful advantages. First, these models avoid systematically taking the dominant category as a reference and the yardstick against all “other” groups is measured (at least in the random part of the model). Second, they solve the problem of the number of interactions to specify, which increases geometrically, by including identity categories as a level in the model specification which thus reduces the increase in the number of parameters to a linear one. This intersectional multilevel approach is based on the idea that identities create similarities between individuals, which in any case violates the assumption of independence between observations that underpin regression modeling, and which can be considered as context for a system of dominance and power. As Evans et al. (2018, p. 67) explain: “individuals may share something concrete – like a neighborhood – they may also share something abstract, like a common set of social exposures associated with their intersectional social identities.”

Multilevel modeling is also advocated to combine variables located at the individual level with organizational or national level variables, due to its potential to analyze identities in relation to wider structures of inequalities (Bauer et al., 2021; Spierings, 2012). Certain groups are systematically disadvantaged through the dominance structure that defines inequalities, with variation in different national contexts: “Analyzing gender relations, or social structures more generally, means focusing on social relations between and among groups of women and groups of men, and on the way the broader social context that constrains and enables individual agents” (Weldon, 2006, p. 238). The increased use of a multilevel approach has demonstrated the limitations of analyzing the effects of different identities (Merlo, 2018), and instead called for a recognition that:

intersectionality does not situate the problems associated with particular identities within individuals or the identities themselves, but within structural power hierarchies, social processes and social determinants that shape the social experiences of individuals with those intersectional identities. While categorical variables (gender, race, class) may be used in regression models, care should always be taken to recognize that these may be intended as proxies for the interactions of systems of oppression (sexism, racism, classism) and other social processes in producing population-level incidence. (Evans et al., 2018, p. 65)

Intersectionality is therefore necessarily structural, and the use of multilevel models allows for interactions between national/institutional variables and individual ones which palliate to the lack of attention paid to how institutions and actors relate to each other but also how identities might not operate in an identical manner across cities, regions, or countries (Hancock, 2007).

The emphasis on the structural aspect of intersectionality is obvious in the words of Anthias, who explains that:

Different modes for the classification of populations, differential treatment on the basis of labelling or attributions of capacities and needs, and modes of exclusion that operate on this basis (the core features of what may be called social divisions) are characteristic of modern social formations. […] Such social divisions permeate societies in different ways although they are by no means universal in the forms they take or in the meanings that underlie the entities constructed. (Anthias, 1998, p. 506)

For Anthias, gender and other characteristics are not only attributes that intersect, they also operate within a wider structure of power relations and inequalities shaped by (a) experience; (b) intersubjective practices and performance; (c) social organization; and (d) social representation. This speaks directly to the need to incorporate a multilevel analytical strategy for gender-based violence in the context of universities and other research organizations, as it recognizes how gender and other characteristics shape (a) individual experiences of different forms of (gender-based) violence and their consequences; (b) how these are enacted relationally as victims, perpetrators, or bystanders; (c) how this is influenced by organizational cultures, settings, contexts, and policies; and (d) the different meanings and frames used to understand and address (gender-based) violence at the national level. Not taking an intersectional approach might mean that beyond not understanding how prevalence might vary across different groups, the institutional measures put in place to combat gender-based violence might not be inclusive and respond to the needs of diverse groups of people (Colpitts, 2022; Voth Schrag, 2017).

Thus far, we have discussed the intersectional lens we feel is needed to better understand gender-based violence, in that it can both shed light on and address potential vulnerabilities not merely by identifying which are the “risky identities” (Merlo, 2018), but by analyzing intersectional factors in relation to potential causal pathways at different levels (Evans et al., 2018). Next, we extend this discussion by integrating the well-known ecological model approach to (gender-based) violence, so that it can better inform our understanding of how the context shapes experiences of gender-based violence, and what factors to consider in an intersectional and contextual multilevel analysis.

## The Importance of Context in Measuring Gender-Based Violence

### Building an Understanding of the Origin of Gender-Based Violence Through Context

The global and universal nature of gender-based violence has been noted—gender-based violence is an “*interclass, intergenerational, intercultural reality*” (Musso et al., 2020, p. 260)—and calls have been made for empirical assessments through cross-national research. However, most studies have a unitary geographical scope, typically a single country or a two–three country comparative element when they go beyond this (Bradbury-Jones et al., 2019). Prevalence rates vary, but there are only limited studies that set to account for these variations across countries or contexts (Heise & Garcia-Moreno, 2002; Humbert et al., 2021; Karlsson et al., 2020; O’Connor et al., 2021).

Gender-based violence is a complex problem, which cannot be explained nor addressed without considering how individual experiences relate to a wider context (Heise, 1998; Wemrell et al., 2019). To understand this, the ecological model originally attributed to Heise (1998) has been influential. According to Heise (1998, p. 262), violence cannot be understood without looking at the “*interplay among personal, situational, and sociocultural factors.*” This ecological model is an attempt to reach an etiology of gender-based violence—that is a study of its causation or origin—using a multilevel social ecology framework, in addition to making the relational character of violence visible so that it can inform effect measures to combat gender-based violence.

**Figure 1.**
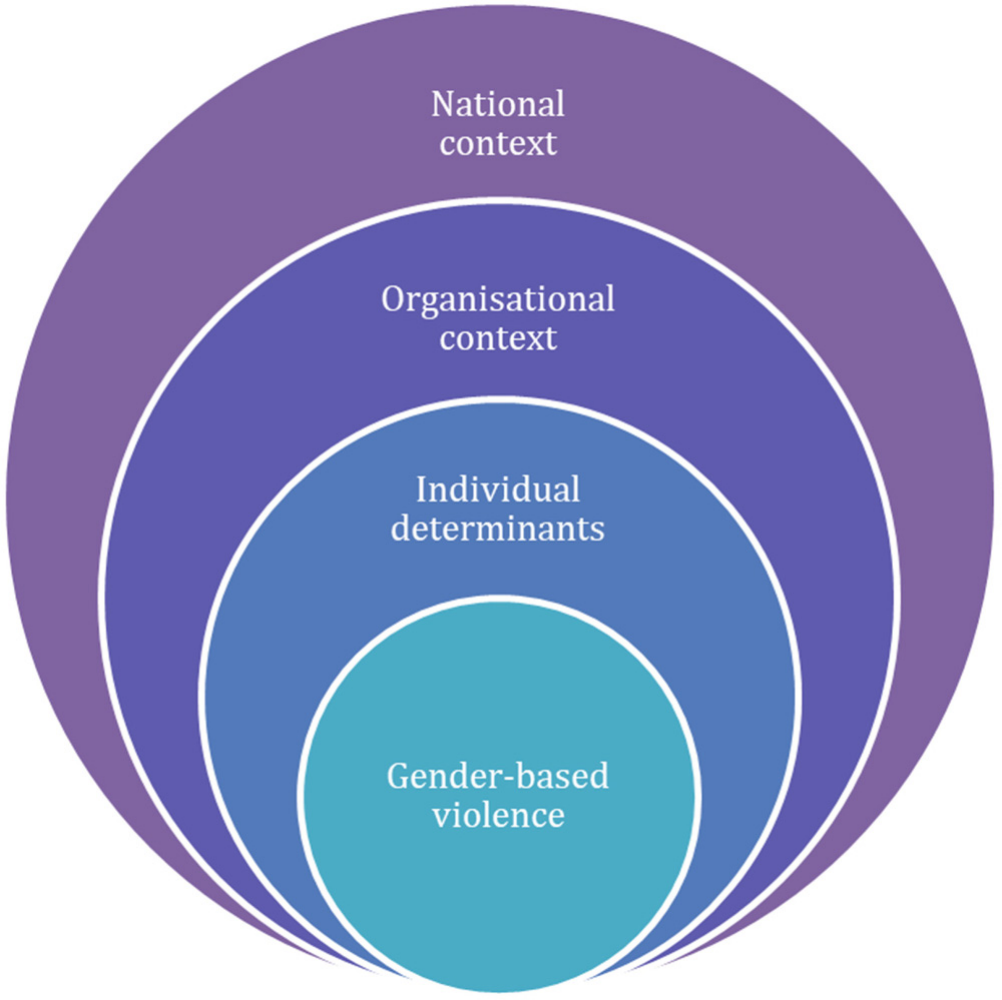
Multilevel factors affecting gender-based violence.

Source: Adapted from Heise (1998).

We adopt this multilevel approach (Figure 1) in an attempt to move from the problems associated with “*single-factor theories [and instead aiming at] explanations that reflect the full complexity and messiness of real life*” (Heise, 1998, p. 262). The ecological model recognizes the importance of understanding how experiences of gender-based violence are nested within interconnected layers (O’Connor et al., 2021). It addresses the problems associated with the limited geographical scope of studies as well as integrates a broader range of factors which hitherto have tended to be skewed toward the individual level as opposed to the community or societal level (Heise & Garcia-Moreno, 2002). Broadening out to incorporate further contextual factors in conjunction with individual ones matters for an etiology of gender-based violence because, as Musso et al. (2020, p. 260, emphasis in the original) argue, “*it has proved vain to seek out individual factors which alone explain the occurrence or non-occurrence of [gender-based violence], and the same is true for contextual or circumstantial factors.*”

### Transposing Theory and Concepts to Measurement of Gender-Based Violence

The challenge is of course access to data that can translate these theoretical and conceptual factors into empirical measures, to test and analyze how different factors—and their interactions across different levels—relate to the prevalence of gender-based violence. This raises the question of the selection of factors—methodological, institutional, or national—that need to be incorporated. This selection needs to be informed by theoretical and conceptual considerations. Heise proposes a number of macrolevel factors (1998, pp. 277–282): the notion of masculinity; rigid gender roles; a sense of men's entitlement/ownership over women; approval of physical chastisement of women; and a cultural ethos that condones violence as a means to settle interpersonal disputes. Other studies have analyzed the relationship between gender beliefs and gender-based violence, with evidence that higher prevalence is associated with more traditional beliefs (Neves et al., 2018), the propensity to blame victims and the acceptance of modern myths particularly in relation to sexual violence (Milesi et al., 2020). At the macrolevel, gender power relations can be understood as a central etiology of gender-based violence, yet this theoretical understanding fails to explain why some (mostly) men are perpetrators of gender-based violence and others are not.

Additional factors include the organizational context to provide a better understanding of how organizational cultures shape the prevalence and consequences of gender-based violence, particularly in relation to universities and other research organizations. O’Connor et al. (2021) also drawing on the work of Bondestam and Lundqvist (2020) and that of Naezer et al. (2019), offer three main organizational features that can worsen the problem of gender-based violence in that specific context. First, hierarchical structures are dominated by men particularly at the top, with low gender egalitarian cultures, and where there is low job satisfaction and engagement. Second, organizations where neoliberal managerialist ethos prevails, often characterized by toxic academic masculine practices fueled by unhealthy competitions for publications and funding and a high reliance on precarious forms of employment. Third, gender/intersectional incompetent leadership, with leadership that fails to take an active stance against gender-based violence, and more generally makes little effort to recruit, retain, or promote women or other actions toward gender structural change. Those and other contextual factors can add to the potential vulnerabilities of people interacting within these contexts.

In practical terms, it has to be acknowledged and recognized that it is not always simple to decide at what level factors ought to be placed. As Heise (1998, p. 266) stresses, “*More important that the location of any single factor is the dynamic interplay between factors operating at different levels. A nested ecological framework explicitly emphasizes the interaction of these factors in the etiology of abuse.*” Various studies have implemented multilevel analysis of gender-based violence empirically. For example, Humbert et al. (2021) examined the effects of methodological, personal, situational, and sociocultural factors on violence against women in the EU. Similarly, Karlsson et al. (2022) included the level of gender equality across the EU in relation to attitudes and beliefs toward gender-based violence, including perceptions of severity and victim-blaming.

While the ecological model is comprehensive in explaining gender-based violence, Heise and Garcia-Moreno (2002) outline two major limitations. First, the factors included in the ecological model need to be recognized as incomplete, and to a large extent purposive (i.e., the tendency to rely on what we can measure, as opposed to what we ought to). Second, the theoretical causality implied in the ecological model also needs to be questioned empirically in terms of asking whether the factors that are considered are truly causal ones, or whether they simply spuriously correlate with incidents of gender-based violence. Another limitation, raised by Cislaghi and Heise (2019, p. 618) recognizes that “*human action almost never originates from a single cause,*” and therefore than a more dynamic framework is needed where domains are allowed to overlap and interact with each other. Nevertheless, we find that the nested structure of the original ecological framework is highly relevant, though we do recognize the mutually shaped constitutions of the different levels of measurements that ought to be considered, and incorporated into an intersectional and contextual multilevel analysis.

## Conclusion

Gender-based violence has consequences at the individual, institutional, and societal levels, though knowledge about the extent of the problem within universities and other research organizations is limited. Eradicating gender-based violence in this context cannot be achieved without knowing the prevalence of gender-based violence. Such measurement allows for testing various theoretical claims that provide insights into why gender-based violence happens in the first place. As Walby et al. (2015, p. 1207) put it, it is important in doing so to “specify the precise forms of inequality, the gendered groups affected, the situations, contexts, practices and mechanisms involved” and to “distinguish between the gender of the victim and gender-saturated contexts […] in which gender-based violence occurs.”

In this article, we have argued that to better understand gender-based violence in the context of universities and other research organizations, it is necessary to ensure that analyses are both intersectional and contextual. The tension and apparent contradiction between intersectionality theory and cross-cultural survey methodology need to be resolved to provide better insights into the heterogeneous experiences of gender-based violence (Ivert et al., 2019). This challenge is further amplified by the emergence of intersectionality theory as a part of Black and antiracist feminist theorizing (Collins, 1990; May, 2015; The Combahee River Collective, 2014). The methodological tensions are evident: quantitative research aims for generalizable findings, which conflict with the nuanced, granular, and specific experiences that intersectionality theory seeks to illuminate. Consequently, the meaning behind any quantified intersections may seem elusive. Furthermore, these meanings can vary across contexts, including how identities are experienced and interpreted across countries.

Despite these challenges, we see value in the temporary stabilization of categories for the pragmatic goal of instigating change. While these categories are not perfect, some knowledge is better than none. We also emphasize the importance of practicing ethical reflexivity—constantly reminding ourselves of our aim to transform the world and seek social justice, and applying this perspective to all facets of our work. Our use of intersectional multilevel modeling, and our application of the MAIHDA approach, is central to that effort and allows us to improve our models by incorporating not just individual characteristics but also modeling intersectional strata, within institutions and countries (Evans, 2019).

To achieve this goal of an analysis that is both intersectional and contextual, our approach also goes beyond a binary understanding of gender and reaches beyond narrow legalistic definitions of gender-based violence (O’Connor et al., 2021). We have offered reflections as to how to operationalize this to derive starting points for intersectional categories to consider and contextual factors to measure at the micro-, meso-, and macrolevels. The application of multilevel intersectional analysis should lead to more nuanced knowledge on experiences of gender-based violence, and thereafter better inform the development of measures to eradicate the problem in an inclusive way.

Understanding the etiology of gender-based violence is necessary to put systems into place to prevent it. Gender-based violence is indeed preventable:

Violence can be prevented and its impact reduced, in the same way that public health efforts have prevented and reduced pregnancy-related complications, workplace injuries, infectious diseases, and illness resulting from contaminated food and water in many parts of the world. The factors that contribute to violent responses – whether they are factors of attitude and behaviour or related to larger social, economic, political and cultural conditions – can be changed. Violence can be prevented. This is not an article of faith, but a statement based on evidence. Examples of success can be found around the world, from small-scale individual and community efforts to national policy and legislative initiatives. (Dahlberg & Krug, 2002, p. 3)

We must acknowledge that addressing gender-based violence is about leveraging the knowledge gained about it and its etiology, to explore the different interventions that can be implemented to address it.

We take inspiration from the application of the ecological model to implement effective interventions (Krug, Dahlberg, et al., 2002), and derive the following implications to inform our future work. First, since gender-based violence is predictable and preventable, a greater understanding of intersectional factors associated with gender-based violence can lead to better interventions. Second, gender-based violence happens in context: understanding this context in relation to gender-based violence is crucial to tailor interventions. Third, gender-violence is autotelic: different forms of gender-based violence correlate with each other, though interventions tend to be fragmented and do not consider how they might be made more effective when implemented in a more integrated manner. Fourth, gender-based violence disproportionately affects “vulnerable” groups: the position of some groups exposes them to gender-based violence more than others, though they often remain invisible and neglected. Interventions focusing on groups at higher risks need to be recognized as an opportunity to address gender-based violence not only for these groups but also for the benefit of all. Fifth, gender-based violence feeds from complacency: the idea that gender-based violence has always existed, just as gender inequalities, perpetuate gender-based violence. Damaging is the idea that gender-based violence is an individual matter, and not for institutions to address, demonstrating the need to examine gender-based violence in the context of organizations. Sixth, and finally, the eradication of gender-based violence needs the commitment of senior leaders within this organizational context: this commitment should include creating research-based evidence and measurement, putting into place adequate policy and legislative frames, funding interventions, increasing awareness and the visibility of the problem as well as the legitimacy of actions to combat it, and ensuring the successful and meaningful implementation of interventions. Measuring and analyzing gender-based violence intersectionally and in context is in fact essential to design and put in place inclusive and structural interventions.
